# Metformin mitigates aortic valve degeneration in an ex vivo three-dimensional tissue model

**DOI:** 10.1038/s41598-025-31070-6

**Published:** 2025-12-17

**Authors:** Friederike Irmgard Schoettler, Andreas Weber, Vera Schmidt, Sebastian Johannes Bauer, Moritz Benjamin Immohr, Artur Lichtenberg, Payam Akhyari, Mareike Barth

**Affiliations:** 1 Department of Cardiovascular Surgery, Medical Faculty, University Hospital Düsseldorf, Heinrich Heine University Düsseldorf, Düsseldorf, Germany; 2 Present Address: Department of Thoracic and Cardiovascular Surgery, Medical Faculty, West German Heart and Vascular Center, University of Duisburg- Essen, Hufelandstr. 55, Essen, 45147 Germany

**Keywords:** Metformin, Aortic valve degeneration, Calcific aortic valve disease, Valvular interstitial cells (VIC), AMPK signaling, Cardiovascular biology, Cardiovascular diseases, Valvular disease, Translational research

## Abstract

Calcific aortic valve disease (CAVD) is a prevalent valvular disorder that lacks effective pharmacological treatments. Metformin, an oral anti-diabetic drug, has been suggested to protect valvular interstitial cells (VICs) from calcification, but its role in preventing organic phosphate-induced degeneration in VICs and aortic valve (AV) leaflets remains unclear. Using an ex vivo three-dimensional tissue model, we induced degeneration with organic phosphate-rich conditions in ovine VICs and AV leaflets. Metformin treatment reduced calcium deposition, as assessed by alizarin red staining (VICs: *p* < 0.0001; AV leaflets: *p* < 0.05), and preserved reduced opacity of AV leaflet (*p* < 0.01). It inhibited early osteogenic differentiation, evidenced by reduced alkaline phosphatase activity (VICs: *p* < 0.0001; AV leaflets: *p* < 0.05), and mitigated gene expression of myofibroblastic markers. Furthermore, metformin improved proliferation (VICs: *p* < 0.0001; AV leaflets: *p* < 0.05), lowered lactate dehydrogenase levels (VICs: *p* < 0.01; AV leaflets: *p* < 0.001), preserved extracellular matrix architecture, and ameliorated extracellular matrix remodeling at gene expression level. AMPK phosphorylation was increased in both VICs (28%, *p* < 0.001) and AV leaflets (21%, *p* < 0.05) under pro-degenerative conditions and metformin treatment. In conclusion, metformin protects against organic phosphate-induced degeneration in both VICs and AV leaflets, highlighting its therapeutic potential in CAVD.

**Supplementary Information:**

The online version contains supplementary material available at 10.1038/s41598-025-31070-6.

## Introduction

Calcific aortic valve disease (CAVD) is the globally most common valvular disorder and a significant cause of aortic stenosis^1,2^. Prevalence and incidence escalate with age, presenting an increasing healthcare burden in our ageing society^3,4^. The underlying active yet gradual multifactorial pathogenesis influences extracellular matrix (ECM) homeostasis and initiates numerous cellular modifications, which trigger fibrotic leaflet thickening prior to widespread calcification and severe degeneration^5–8^.

Aortic valves (AVs) consist of semilunar leaflets with three layers: the fibrosa, the spongiosa, and the ventricularis^7,9^. Valvular interstitial cells (VICs) are present in all three AV layers^5^ and are essential players in the progression of CAVD^10^. Therein, quiescent VICs are assumed to proceed to fibrotic changes and calcification by differentiating into activated, myofibroblastic and osteoblastic-like phenotypes^11^. Myofibroblastic differentiation is characterized by an upregulation of alpha smooth muscle actin expression and simultaneous vimentin downregulation^10,12^. Osteoblastic differentiation is characterized by an early enhancement of alkaline phosphatase (ALP) activity, runt-related transcription factor 2, and bone morphogenetic protein 2 upregulation, as well as osteopontin and osteocalcin expression^10,13^. Ultimately, calcium deposition is the specific indicator of late stages of calcific degeneration^10^.

To date, we lack pharmacologic treatment options for CAVD^14^. The primary available treatment options for CAVD are surgical and transcatheter aortic valve replacement^14^. However, recent investigations have unveiled that patients usually already suffer severe symptoms chaperoned by myocardial remodeling prior to valve replacement^15,16^. Clinical studies have been initiated to lead the path to pharmacologic treatment options for CAVD, however, not yet presenting an effective agent^15,17^. Hence, there is still a pressing need for pharmacological approaches aimed at CAVD prevention and mitigation.

Metformin is a biguanide derivate and the most frequently used oral antidiabetic as first-line therapy for type 2 diabetes mellitus^18^. In hepatocytes, metformin actively enters the cell via organic cation transporter 1^19,20^. It directly inhibits mitochondria complex I, consequently, increasing AMP/ATP and ADP/ATP ratios by preventing ATP production, thus activating AMP-activated protein kinase (AMPK)^21^. Recent evidence has also linked AMPK activation to mitigation of tissue fibrosis and degeneration via inhibition of myofibroblast differentiation as well as prevention of vascular calcification^22,23^. Metformin was previously found effective as an off label used therapeutic and linked to cardiovascular conditions^24,25^. Specifically in the context of CAVD, recent studies suggested that metformin detains VIC calcification in inorganic phosphate-induced two-dimensional (2D) cell culture calcification models by PI3K/AKT pathway activation in an AMPK dependent manner^26^ and by initiating autophagic flux^27^. To enable a future translation to clinical implications, these findings warrant further validation under conditions that are closer to in vivo conditions, particularly incorporating (1) organic phosphate and (2) VIC in a native-like three-dimensional (3D) ECM environment. Therefore, we investigated whether metformin reliably protects against organic phosphate-induced degeneration in both VICs and AV leaflets using an ex vivo 3D CAVD tissue model.

## Results

### Metformin mitigates the degeneration of valvular interstitial cells (VICs) and aortic valve leaflets

**Fig. 1:**
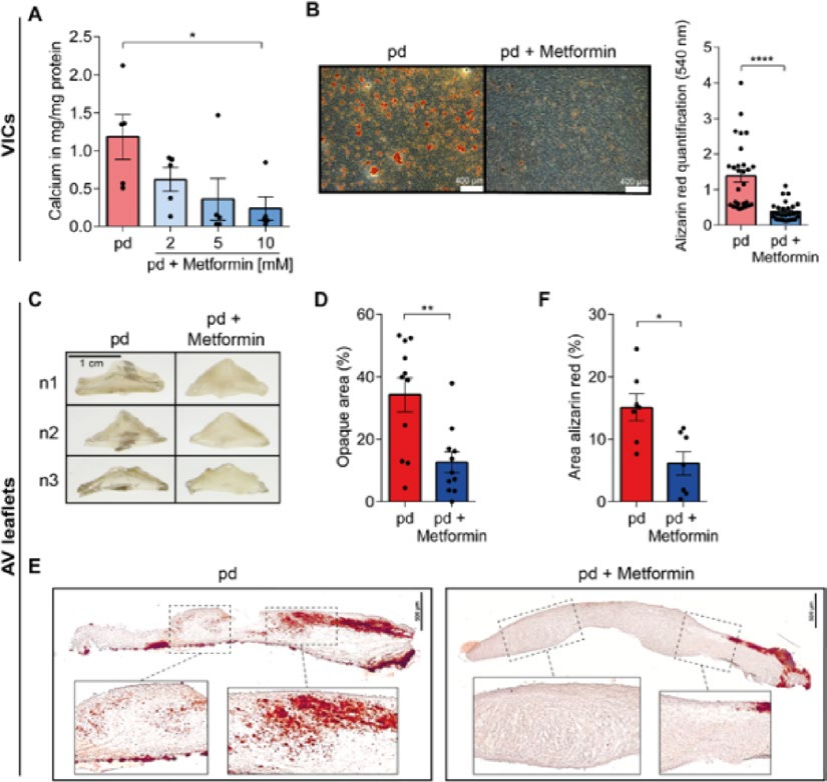
Metformin inhibits degeneration of valvular interstitial cells (VICs) and aortic valve (AV) leaflets. **A** Calcium content in VIC cultures determined by colorimetric assay and normalized to protein content in pd and pd + metformin treatment (n = 5). **B** Representative microscopic images (left) and quantification of alizarin red S calcium staining of treated VICs (*right*). (n = 28) Scale bar = 400 µm. **C** Representative macroscopic images of treated AV leaflets. Scale bar = 1 cm. **D** Quantification of opaque area of treated AV leaflets presented as percentage of total AV leaflet area. (n = 11). **E** Representative microscopic image of alizarin red S calcium staining of treated AV leaflets. Scale bar = 500 µm. **F** Quantification of alizarin red S calcium stained areas as percentage of total AV area (n = 7). p-values were calculated by using either nonparametric Kruskal-Wallis-test with Dunn’s post hoc correction (**A**) or nonparametric Mann-Whitney-U test (**B**, **D**, **F**). Data are presented as mean ± SEM. * = p < 0.05, ** = p < 0.01, **** = p < 0.0001. *AV – aortic valve, µM – micrometer, pd – pro-degenerative, VIC – valvular interstitial cell*.

To determine the optimal concentration of metformin for VIC cultures, we measured calcium deposition per total protein amount under organic phosphate pro-degenerative (pd) conditions across a range of metformin concentrations from 2 to 10 mM (Fig. 1A). Here, a concentration of 10 mM metformin significantly reduced calcium deposition (*p* = 0.0446) which could be also confirmed by alizarin red S calcium staining and quantification (Fig. 1B, *p* < 0.0001; Supplementary Fig. S1 online); thus, this concentration was used for further experiments. Having established the anti-degenerative effects of metformin in VIC cultures, we sought to explore whether these protective effects extend to AV leaflets. Therefore, we utilized our recently established in vitro CAVD tissue model^28,29^ and treated cultured AV leaflets under pd-conditions with metformin. Metformin mitigated the degeneration of AV leaflets cultured under pd-conditions for 28 days, as shown by a significant reduction of opaque areas by 63%, indicative for thickening of the leaflets due to fibrosis and calcification (Fig. 1C + D; *p* = 0.0052). Furthermore, alizarin red S calcium staining revealed diminished calcium accumulation under metformin treatment in both the ventricularis and the fibrosa layer compared to controls (Fig. 1E). Quantification of alizarin red S calcium stained areas further confirmed the anti-degenerative effect of metformin in AV leaflets (Fig. 1F, *p* = 0.0175). Extension of the cultivation duration of AV leaflets under pd-conditions and metformin treatment up to 56 days supported these findings and underlined metformin’s protective ramifications in long-term cultures (Supplementary Fig. S2 online). Herein, opaque areas are reduced (Supplementary Fig. S2A + B online, *p* = 0.0317) and calcium accumulation is diminished by metformin treatment, as indicated by alizarin red S calcium staining and quantifications (Supplementary Fig. S2C + D online, *p* = 0.0002). Taken together, these results suggest metformin as a potent inhibitor of calcium accumulation under pd-conditions in both VICs and whole AV leaflets.

## Metformin decreases alkaline phosphatase activity in both VICs and AV leaflets

**Fig. 2:**
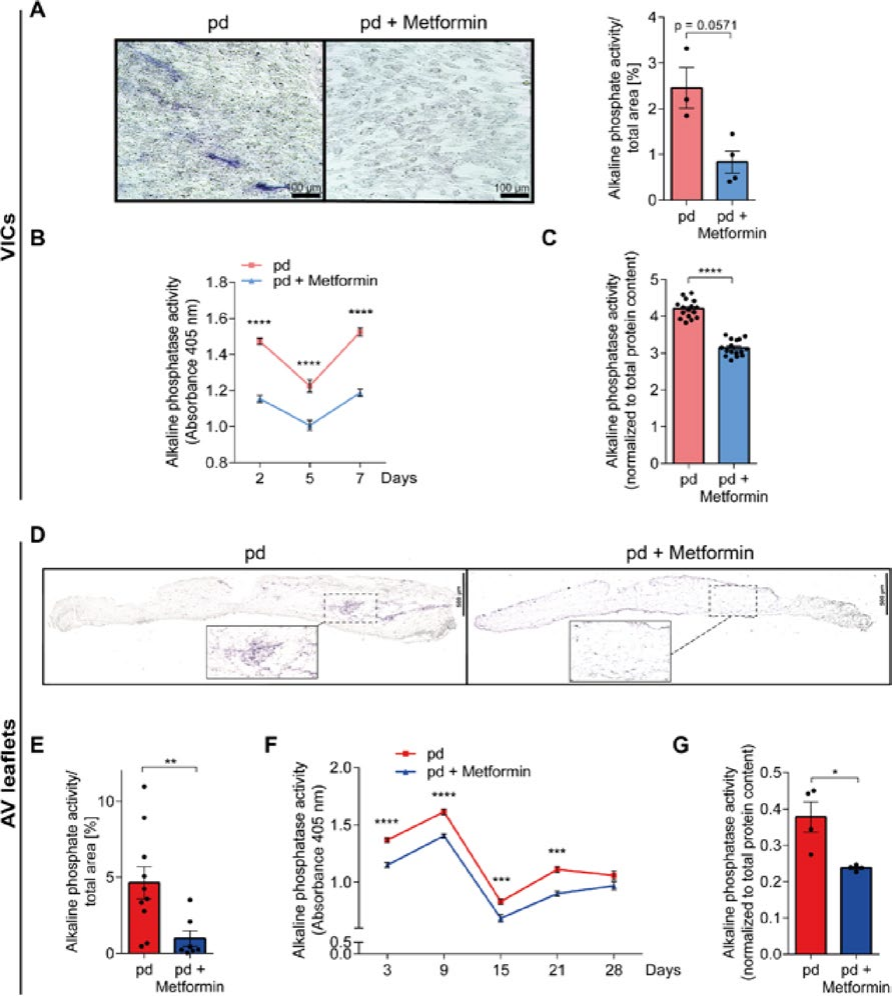
Metformin decreases alkaline phosphatase (ALP) activity in both VICs and AV leaflets. **A** Representative microscopic images of ALP activity staining in VICs treated with pd and pd + metformin (left). Scale bar = 100 µm. Quantification of ALP staining (n = 3 – 4, right). **B** ALP activity in VIC culture supernatants at day 2, 5, and 7. **C** ALP activity in VIC cultures normalized to total protein content at day 7 (n = 16). **D** Representative microscopic images of AV leaflets stained for ALP activity. Scale bar = 500 µm. **E** Quantification of ALP-stained areas shown as percentage of total leaflet area (n = 7 – 10). **F** ALP activity in AV leaflet culture supernatants at day 3, 9, 15, 21, and 28 (n = 7 – 13). **G** ALP activity of AV leaflets normalized to total protein content at day 28 (n = 4). p-values were calculated by using either nonparametric Mann-Whitney-U test (**A**, **C**, **E**, **G**) or two-way analysis of variance (ANOVA) with Sidak’s post-hoc test (**B**, **F**). Data are presented as mean ± SEM. * = p < 0.05, ** = p < 0.01, *** = p < 0.001, **** = p < 0.0001. *ALP – alkaline phosphatase, AV – aortic valve, µM – micrometer, pd – pro-degenerative, VIC – valvular interstitial cell*.

Alkaline phosphatases (ALP) hydrolyse inorganic pyrophosphate and produce inorganic phosphate, thus promoting biomineralization. To assess for present biomineralization and early osteogenic differentiation, we examined ALP activity in both VIC cultures and AV leaflet tissue cultures. Initially, we investigated the activity in VIC cultures using in-well ALP staining (Fig. 2A, *left*). While quantification of areas stained for ALP did not reach statistical significance, it suggested a trend towards reduced ALP activity by metformin treatment (Fig. 2A, *right*, *p* = 0.0571). Subsequently, we measured the ALP activity in cell culture supernatants and detected significantly decreased total ALP activity by metformin treatment compared to pd-controls for all examined timepoints (Fig. 2B days: *p* < 0.0001; 5 days: *p* < 0.0001; 7 days: *p* < 0.0001). At day 7, cellular ALP activity normalized to total present protein content was also significantly reduced by metformin treatment (Fig. 2C, *p* < 0.0001). Next, we investigated whether metformin similarly decreases ALP activity in AV leaflets, thus inhibiting tissue biomineralization. We performed histological ALP staining of AV leaflets (Fig. 2D). Quantification of stained areas revealed significantly reduced ALP activity mediated by metformin treatment compared to pd-controls (Fig. 2E, *bottom*, *p* = 0.0031). Metformin treatment under pd-conditions also decreased the ALP activity in AV leaflet culture supernatants (Fig. 2F; 3d: *p* < 0.0001, 9 d: *p* < 0.0001, 15 d: *p* < 0.001, 21 d: *p* < 0.001, 28 d: *p* = 0.1007). At 28 days, normalization of ALP activity in conditions culture media from AV leaflets to total protein content of the tissue was significantly reduced by metformin treatment as compared to pd-controls (Fig. 2G, *p* = 0.0286).

## Metformin modulates the expression of genes involved in osteogenic differentiation

**Fig. 3:**
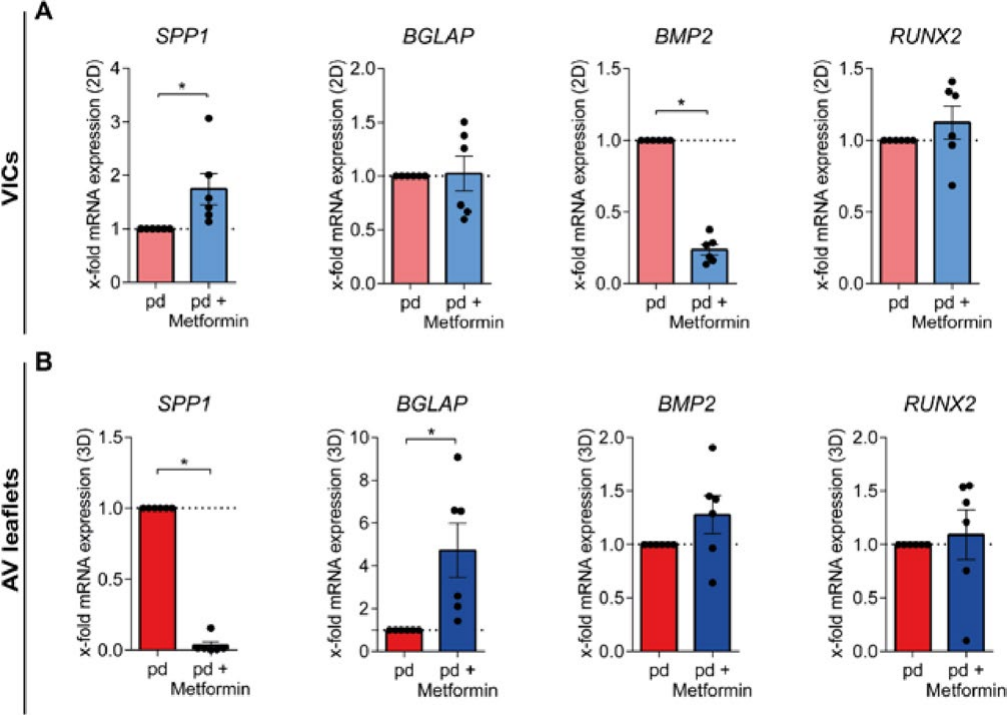
Metformin modulates the expression of genes involved in osteogenic differentiation. Gene expression analysis of osteogenic markers of VIC cultures **(A)** and AV leaflets **(B)**. Relative x-fold gene expression of *SPP1*, *BGLAP*, *BMP2*, and *RUNX2* (*n* = 6). p-values were calculated by using nonparametric Wilcoxon-signed-rank test. Data are presented as mean ± SEM. * = *p* < 0.05. *AV – aortic valve, BMP2 – bone morphogenic protein 2, BGLAP – osteocalcin, SPP1 – osteopontin, pd – pro-degenerative, RUNX2 – runt-related transcription factor 2, VIC – valvular interstitial cell*.

Gene analysis was performed to investigate the impact of metformin treatment on the expression of genes involved in osteogenic differentiation in both VICs and AV leaflets. In VICs, the expression of osteopontin (*SPP1*) was increased by metformin compared to pd-controls (Fig. 3A, *p* = 0.0313). In contrast, the expression of bone morphogenic protein 2 (*BMP2*) was reduced by metformin treatment in VICs (Fig. 3A, *p* = 0.0313). Further, the expression of osteocalcin (*BGLAP*) and runt-related transcription factor 2 (*RUNX2*) was not altered by metformin in VICs (Fig. 3A, *p* = 1.0). However, *BGLAP* expression was enhanced in AV leaflets treated with metformin under pd-conditions (Fig. 3B, *BGLAP*, *p* = 0.0313), while *SPP1* expression was significantly decreased (Fig. 3B, *SPP1*, *p* = 0.0313). Interestingly, *RUNX2* and *BMP2* expression were not altered by metformin treatment in AV leaflets (Fig. 3B, *RUNX2*: *p* = 0.6875, *BMP2*: *p* = 0.2188).

## Metformin reduces proliferation, alters metabolism and differentiation of VICs and AV leaflets

**Fig. 4:**
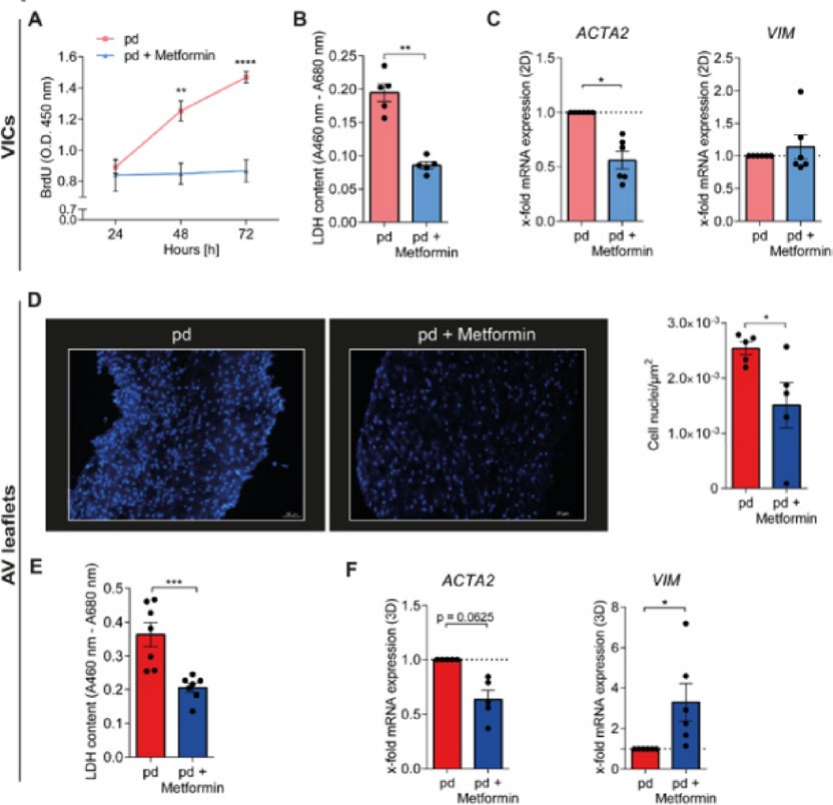
Metformin reduces proliferation, lactate dehydrogenase activity, and alleviates myofibroblastic differentiation in VICs and AV leaflets. **A** Assessment of cell proliferation in VIC cultures using BrdU incorporation for VICs treated with pd and pd + metformin at 24, 48, and 72 hours (h). (*n* = 11–12). **B** LDH content in VIC culture supernatants (*n* = 5). **C** Relative x-fold *ACTA2* and *VIM* gene expression of treated VICs (*n* = 6). **D** Representative microscopic images of fluorescent DAPI staining of AV leaflets (*left*). Scale bar = 50 μm. Quantification of cell nuclei per µm^2^ of DAPI-stained AV leaflets (*right*) (*n* = 5). **E** LDH content in AV leaflet culture supernatants (*n* = 7). **F** Relative x-fold *ACTA2* and *VIM* gene expression of AV leaflets (*n* = 5–6). p-values were calculated by using either two-way analysis of variance (ANOVA) with Sidak’s post-hoc test (**A**), nonparametric Mann-Whitney-U test (**B**,** D**,** E**)or nonparametric Wilcoxon-signed-rank test (**C**,** F**). Data are presented as mean ± SEM. * = *p* < 0.05, ** = *p* < 0.01, *** = *p* < 0.001, **** = *p* < 0.0001. *ACTA2 – alpha smooth muscle actin, AV – aortic valve, BrdU – 5-bromo-2’-deoxyuridine, DAPI - fluorescent 4′,6-diamidino-2-phenylindole, LDH – lactate dehydrogenase, µM – micrometer, pd – pro-degenerative, VIC – valvular interstitial cell, VIM – vimentin*.

Next, we intended to evaluate the effects of metformin on cellular homeostasis and differentiation. Metformin treatment significantly inhibited VIC proliferation after 48 and 72 h (Fig. 4A, 48 h: *p* = 0.0003; 72 h: *p* < 0.0001), respectively. Detection of lactate dehydrogenase (LDH) levels showed reduced LDH content in cell culture supernatants of VICs treated with metformin (Fig. 4B, *p* = 0.0079). Metformin treatment significantly reduced glucose concentrations of VIC cultures treated under pd-conditions for 7 days (Supplementary Fig. S3 online, *p* < 0.0001). Gene expression analysis of alpha smooth muscle actin (*ACTA2*) and vimentin (*VIM*) was conducted to investigate myofibroblastic differentiation. Treatment with metformin decreased the expression of *ACTA2* (Fig. 4C, *p* = 0.0313), while *VIM* gene expression remained unaffected in VICs (Fig. 4C, *p* = 1.0).

Further, we aimed to investigate the impact of metformin on AV leaflet homeostasis and myofibroblastic differentiation. Staining with 4′,6-Diamidin-2-phenylindol (DAPI) of AV leaflets cultured for 28 days under pd-conditions and treated with metformin revealed preserved cellularization in both pd-controls and metformin treated AVs (Fig. 4D, *left*). Quantification analysis of cell count demonstrated less DAPI positive cell nuclei by metformin treatment (Fig. 4D, *right*, *p* = 0.0317). Similarly, as observed for VIC 2D cultures, metformin treatment reduced the LDH content in supernatants compared to pd-controls (Fig. 4E, *p* = 0.0006, Supplementary Fig. S4 online). Also, in line with the results in the VIC cultures, metformin treatment significantly reduced glucose concentrations in AV leaflet culture supernatants measured after 28 days of cultivation (Supplementary Fig. S3 online, *p* = 0.0022). Gene expression analysis showed that metformin treatment reduced the gene expression of *ACTA2* in AV leaflets compared to pd-controls by trend (Fig. 4F, *ACTA2*, *p* = 0.0625), while increasing the gene expression of *VIM* (Fig. 4F, *VIM*, *p* = 0.0313). ACTA2/VIM ratio in 3D AV leaflets was significantly reduced (Supplementary Fig. S5 online, *p* = 0.0317). These results collectively imply a protective impact of metformin on valve homeostasis and suggest an inhibition of myofibroblastic differentiation in AV leaflets as well as an alteration of metabolism in both VIC and AV leaflet cultures.

## Metformin alleviates matrix remodeling and preserves matrix architecture of AV leaflets

**Fig. 5:**
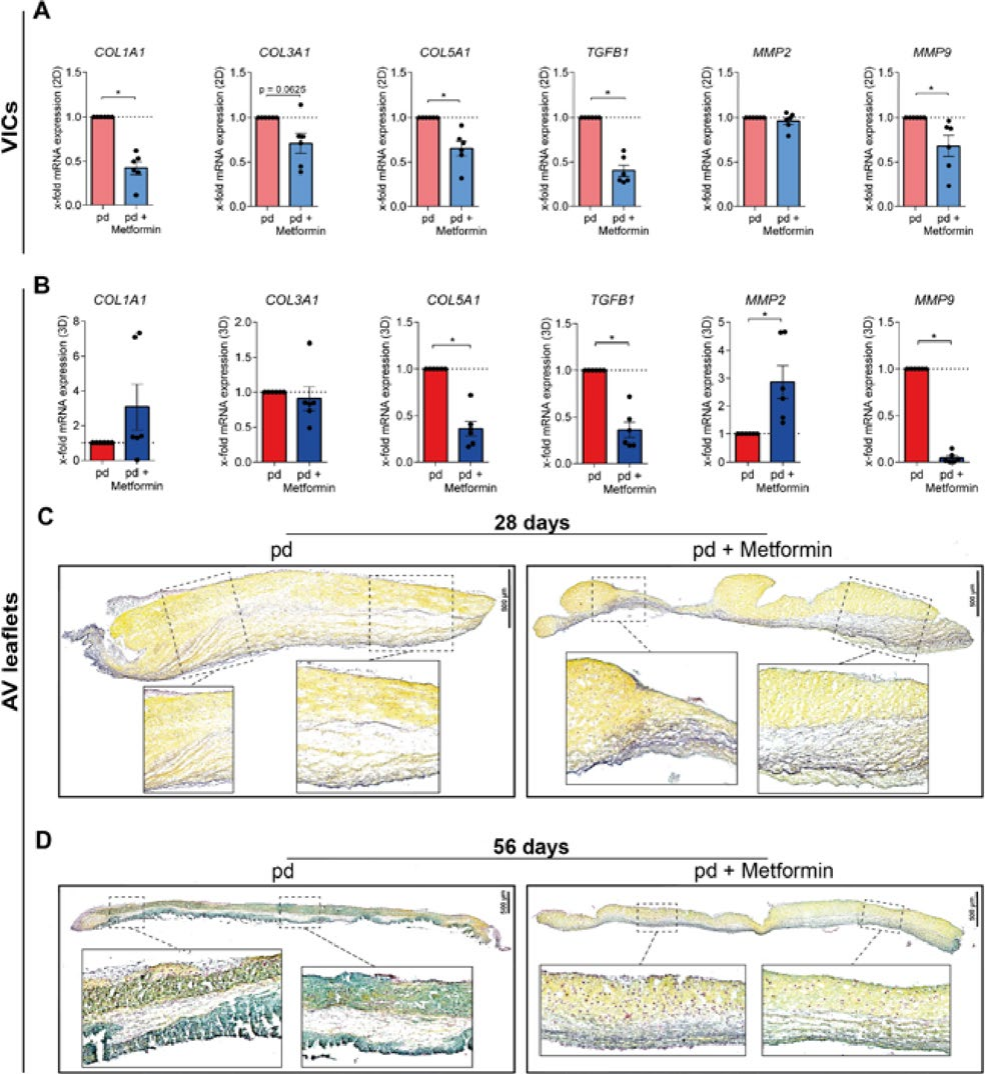
Metformin preserves extracellular matrix (ECM) architecture in AV leaflets and modulates the expression of genes involved in ECM remodeling. Gene expression analysis of ECM markers of VIC cultures **(A)** and AV leaflets **(B)**. Relative x-fold gene expression of *COL1A1*, *COL3A1*, *COL5A1*, *TGFB1*, *MMP2*, and *MMP9*, is shown (*n* = 5–6). p-values were calculated by using nonparametric Wilcoxon-signed-rank test. Data are presented as mean ± SEM. * = *p* < 0.05. Representative microscopic images of Movat’s pentachrome staining of AV leaflets treated with pd and pd + metformin at culture day 28 **(C)** and 56 **(D)**. Scale bar = 500 μm. *AV – aortic valve, COL1A1 – alpha- type I collagen, COL3A1 – alpha- type III collagen, COL5A1 – alpha- type V collagen, ECM – Extracellular matrix, MMP2 – matrix metalloproteinase 2, MMP9 – matrix metalloproteinase 9, µm – micrometer, TGFB1 – transforming growth factor beta 1*.

Next, we investigated the impact of metformin treatment on ECM remodeling using gene expression analysis (Fig. 5A + B) as well as histological staining (Fig. 5C + D). Compared to pd-controls, metformin treatment in VIC cultures significantly reduced the gene expression of type I collagen (Fig. 5A, *COL1A1*, *p* = 0.0313), and *COL5A1* (Fig. 5A, *p* = 0.0313), while a trend in reducing *COL3A1* (Fig. 5A, *p* = 0.0625) was observed. Moreover, metformin treatment decreased the expression of transforming growth factor beta 1 (Fig. 5A, *TGFB1*, *p* = 0.0313) and matrix metalloproteinase (MMP) 9 (Fig. 5A, *MMP9*, *p* = 0.0313) in VICs. *MMP2* was not modulated by metformin in VICs (Fig. 5A, *p* = 0.437). Similarly to VICs, in AV leaflets the gene expression of *COL5A1* (Fig. 5B, *p* = 0.0313), *TGFB1* (Fig. 5B, *p* = 0.0313), and *MMP9* (Fig. 5B, *p* = 0.0313) was significantly reduced by metformin treatment under pd-conditions. Conversely, metformin treatment of AV leaflets increased the expression of *MMP2* (Fig. 5B, *p* = 0.0313) compared to controls, while not significantly influencing the expression of *COL1A1* (Fig. 5B, *p* = 0.0625) and *COL3A1* (Fig. 5B, *p* = 0.4375). To assess modifications in the ECM architecture, Movat’s pentachrome staining was performed (Fig. 5C + D). After 28 cultivation days, pd-conditions result in a thicker collagen-rich (yellow) fibrosa layer and partially disorganized ECM structure compared to metformin treatment (Fig. 5C). Further, after long-term cultivation for 56 days under pd-conditions, the ECM structure appeared disturbed; AV leaflets thickened inconsistently, and all three AV layers were enriched with proteoglycans (blue) (Fig. 5D). Metformin treatment, however, protected the ECM structure and trilaminar architecture of AV leaflets.

### AMPK phosphorylation is induced by Metformin treatment in both VICs and AV leaflets

**Fig. 6:**
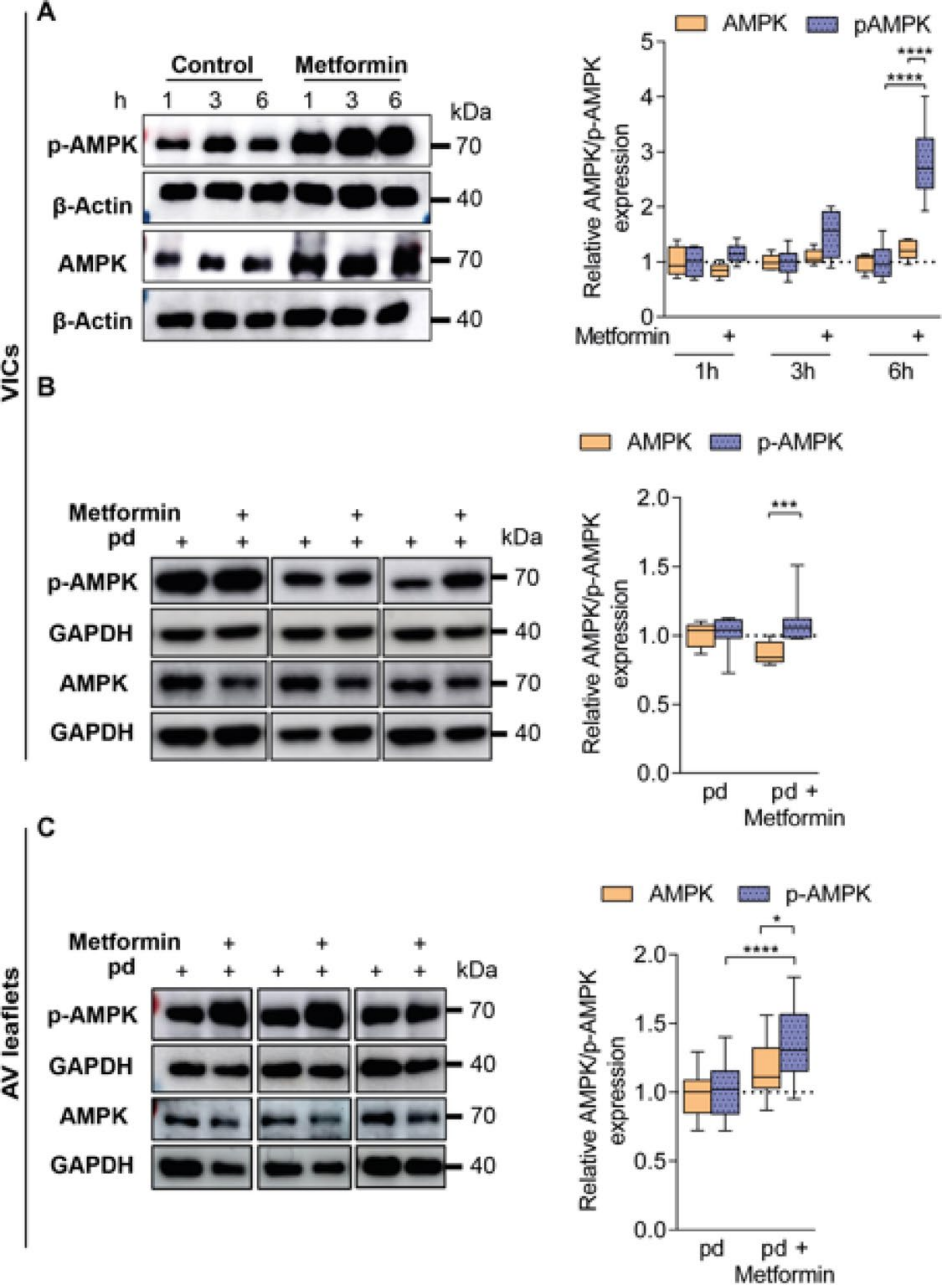
Metformin treatment induces AMP-activated protein kinase (AMPK) phosphorylation in VICs and AV leaflets. **A** Representative cropped Western blot images of VIC cultures (*left*) for AMPK, phosphorylated AMPK (pAMPK), and β-actin at 1, 3, and 6 h under control conditions (10% culture medium) and metformin treatment. Density analysis (*right*) for quantification of relative AMPK phosphorylation, normalized to β-actin (*n* = 6). Uncropped Western blot membrane images are displayed in Supplementary Figure S6. **B** Representative cropped Western blot images of VIC cultures (*left*) for AMPK, pAMPK, and GAPDH after 7 days of cultivation under pd and pd + metformin. Density analysis (*right*) for quantification of relative AMPK phosphorylation, normalized to GAPDH (*n* = 11). Uncropped Western blot membrane images are displayed in Supplementary Figure S7. **C** Representative cropped Western blot images of AV leaflets (*left*) after 28 days of cultivation under pd and pd + metformin for AMPK, pAMPK, and GAPDH. Density analysis (*right*) for quantification of relative AMPK phosphorylation, normalized to GAPDH (*n* = 15). Uncropped Western blot membrane images are displayed in Supplementary Figure S8. p-values were calculated by using two-way analysis of variance (ANOVA) with Sidak’s post-hoc test. Data are presented as mean ± SEM. * = *p* < 0.05, *** = *p* < 0.001. **** = *p* < 0.0001.

For assessment of potential mechanistic pathways contributing to the protective and modulatory effects of metformin, we investigated AMPK phosphorylation. Metformin has been described to induce AMPK signaling, protect against calcification in an AMPK-dependent manner, and partially prevented osteoblastic differentiation by AMPK activation^21,26,30^. To verify whether the AMPK signaling pathway is upregulated in our VIC and AV leaflet cultures, we performed Western blot analysis. Short term incubation with metformin under control-conditions (10% culture medium) for 1 up to 6 h significantly enhanced AMPK phosphorylation in VIC cultures at timepoint 6 h compared to the respective control by 180% and the phosphorylation level was significantly increased in metformin treated VICs (Fig. 6A, control vs. metformin pAMPK at 6 h: *p* < 0.0001; metformin AMPK 6 h vs. metformin pAMPK at 6 h: *p* < 0.0001; Supplementary Fig. S6 online). Further, phosphorylation analysis after 7 days of cultivation under pd-conditions and metformin treatment showed an increased relative AMPK phosphorylation induced by metformin by 28% (Fig. 6B, *p* = 0.0002; Supplementary Fig. S7 online). Similarly to VICs, in AV leaflets cultured for 28 days under pd-conditions, the phosphorylation of AMPK is enhanced under metformin treatment by 21% (Fig. 6C, *p* = 0.0177; Supplementary Fig. S8 online). The phosphorylation level of AMPK was significantly higher in metformin treated AV leaflets compared to pd-controls (Figs. 6C, 38%, *p* < 0.0001).

## Discussion

CAVD imposes a worldwide medical burden, yet no pharmacological treatment options are available^14^. Metformin, the first line oral antidiabetic therapeutic for type II diabetes^18^, has recently been suggested to detain inorganic phosphate-induced calcification of VIC in 2D cell cultures^26^. However, its effects on organic phosphate-induced degeneration in whole AV leaflets remain unclear. This is important, since organic phosphates actively promote calcification by not only providing inorganic phosphate but also promoting hotspots for calcium deposition, which intensifies valve stiffening^31^. Thus, CAVD progression is directly influenced by the dual role of organic phosphates, acting as both source and catalyst for calcium accumulation. Targeting this dual role may provide a novel therapeutic approach for early-stage CAVD. This study provides novel evidence on protective effects of metformin, which are mediated by (1) protection against organic phosphate-induced calcification in both VIC and whole AV leaflets; (2) inhibition of myofibroblastic and early osteogenic differentiation in both VIC and AV leaflets; and (3) preservation of ECM integrity in AV leaflets.

Previously, the importance of culture media conditions for studies on human aortic VIC degeneration and calcification has been emphasized. Human VICs show differences in adapting to inorganic and organic phosphate-rich media, with significant impact on obtained results^32^. This is particularly important and needs to be considered in order to reduce passage-dependent variations in in vitro models^32^. In our study, ovine VICs and AV leaflets were cultured under distinct pd-culture conditions using β-GP as organic phosphate source, which initiates osteogenic shifts by ALP-conditioned degeneration^28,32–34^. Our results show that metformin diminishes organic phosphate-induced calcification in both VICs and AV leaflets, thus proposing metformin as a therapeutic for CAVD based on a clinically applicable ex vivo CAVD tissue model.

Initiation and progression of CAVD involves upregulation of ACTA2 expression and downregulation of VIM, resembling the differentiation of VICs into activated myofibroblasts^10,12,35^. Our data suggests that metformin is capable of inhibiting myofibroblastic differentiation - a hallmark of CAVD progression - in VICs and by trend in AV leaflets, rather keeping the cells’ mesenchymal fibroblastic phenotype^36^. Further, the reduced proliferation rate and consistently diminished LDH secretion under metformin treatment underscore the protective ramifications observed in both VICs and AV leaflets^11^. Reduced LDH content may indicate a metformin-induced reduction in cell damage, suggesting a potential cytoprotective effect. On the other hand, alterations in LDH content could reflect metabolic reprogramming of VICs, as metformin acts through mitochondrial complex 1, thereby affecting pyruvate metabolism and consequently lowering lactate and LDH levels^37,38^. Furthermore, metformin reduces glucose concentrations in both VIC and AV leaflet culture supernatants, thus supporting the notion of metformin-induced metabolic shifts.

Concomitantly with myofibroblastic differentiation, osteoblastic differentiation also contributes to the progression of CAVD^10^. Metformin mitigated early osteogenic differentiation as shown by a reduced ALP activity and alleviated biomineralization by trend in VICs and significantly in AV leaflets. ALP is upregulated in the presence of organic phosphate and plays a pivotal role in the calcification process, whereas an increased ALP activity indicates early osteoblastic differentiation^10,13,39^. The complex mechanisms underlying osteogenic differentiation also involve upregulation of *BMP2*, *RUNX2*, and modulation of *SPP1* and *BGLAP*^10,11,13,40,41^. Previous studies have shown metformin as an inhibitor of inorganic phosphate induced osteoblastic differentiation by decreasing BMP2 and SPP1 at protein level^26^. However, gene expression of osteogenic differentiation markers was not consistently modified in our study: A reversed regulation of *SPP1* with upregulation in VICs and downregulation in AV leaflets by metformin treatment was observed. The *BGLAP* gene expression is increased in AV tissue under pd-conditions treated with metformin, whereas recent studies have shown conflicting results on the regulation of osteocalcin by metformin^42–45^. Further, discrepancies in pro-osteogenic gene expression markers between 2D and 3D culture approaches have been recognized previously^28^. Similar findings have also been observed when comparing static and dynamic 3D models of valvular degeneration^29^. These differences may be attributable to matrix-associated, shear stress-related alterations in gene expression between 2D and 3D cultures. In this context, the effects of metformin are likely mediated by mechanisms including matrix components, as reflected by the diverse gene expression of collagen subtypes and MMPs. These findings underscore the inevitable need of 3D models, since the presence of ECM molecules and thereby different biomechanics may profoundly impact these markers^29^. Moreover, the expression of these markers might be time- or stage-dependent. These aspects make it difficult to directly link the gene expression of markers like *BMP2*, *SPP1*, *BGLAP*, and *RUNX2* to a certain pathological observation. The expression of *TGFB1*, a key regulator in degenerative processes and adapting to tissue damage^46^, was uniformly decreased by metformin in our models. These findings are in line with previous reports on metformin acting as a conditioner of TGF-β1-induced osteoblastic differentiation in human aortic VICs^30^.

Considering the role of ECM, metformin treatment sufficiently protected the ECM integrity in our experiments. CAVD is based on a complex pathogenesis also implementing the interaction and communication of the present cell types with the surrounding ECM^10,11,47^. Therefore, 2D cell monocultures are presumably limited and further complicated by the absence of a physiologic ECM^48,49^. We utilized a previously established CAVD tissue culture model that provides an ECM structure and allows for structural analyses^28,36^. Despite structural criteria, MMPs have been identified as upregulated in CAVD and may accelerate degenerative processes by encouraging calcific and fibrotic ECM remodeling^50,51^. Specifically, MMP9 is secreted by activated VICs and was found to be enhanced in aortic stenosis^50,51^. Thus, the herein described finding on reduced *MMP9* expression in both VICs and AV leaflets support the protective character of metformin treatment. Interestingly, *MMP2* remains unaltered by metformin in VICs but exhibits upregulation in AV leaflets. Although *MMP2* upregulation could initially suggest a pro-degenerative stimulus, we contend that it signifies the intricate nature of CAVD pathogenesis. Further, cusp specific differences for the calcification potential of aortic valves were previously described^52^. The protective ramifications of metformin on ECM remodeling are further supported by modulation of collagen expression^7^. Metformin treatment resulted in a uniformly diminished expression of all examined collagens in VICs in this study. In AV leaflets, we recently discovered that the gene expression of collagens is enhanced after 28 days of pd-conditions^28^. In the present work, metformin treatment decreased the expression of *COL5A1* in AV leaflets. On the other hand, *COL1A1* expression was by trend increased. It should be considered that up to approximately 70% of the physiological AV collagen matrix consists of *COL1A1*^53,54^. Collagen organization can be assessed using Sirius Red staining; however, this approach does not permit discrimination between distinct collagen subtypes. Nevertheless, in-depth analyses of specific collagen remodeling processes incorporating Sirius Red staining together with the assessment of collagen hydrolases are planned for future investigations. Recent evidence suggests gender-specific differences in fibrocalcific and ECM remodeling^55^. Thereby, an enhanced myofibroblastic remodeling was found in male VICs, while female VICs showed a higher collagen production, MMP-levels, and metabolic activity. This is particularly important as our study only includes male ovine species and did not assess for a gender-specific impact.

Mechanistically, metformin activates AMPK, which is known to coordinate cell growth and autophagy^21,56^. Previous studies suggested that metformin detains calcification in VICs in an AMPK-dependent manner and alleviates osteoblastic differentiation through AMPK activation^26,30^. In the present study, the addition of metformin to pd-conditions led to a significantly increased AMPK phosphorylation in both VICs and whole AV leaflets. This underlines the AMPK signaling pathway as a potential key player in the mechanism of action of metformin also in 3D tissue cultures. In fact, metformin induces multiple metabolic pathways via AMPK phosphorylation, including autophagy and cell growth by inhibition of the mammalian target of rapamycin complex 1^56–58^. Autophagic flux induced by metformin treatment was recently shown to alleviate calcification in rat VICs^27^. Our findings presented here, along with the aforementioned previously published data highlight metformin’s multiple, and potentially beneficial mechanism of action, which could favor its utilization as therapeutic for CAVD.

Given metformin’s widespread use in diabetes type II and hyperglycemia, which are common risk factors for CAVD, it may already benefit predisposed patients. Additionally, metformin’s clinical relevance is supported by its off-label use in conditions such as polycystic ovary syndrome and gestational diabetes, suggesting a potentially broader therapeutic role^24^. The presented multifaceted ramifications on calcification, differentiation, and ECM remodeling position metformin as a potential therapeutic for CAVD.

## Conclusions

Metformin significantly diminishes degenerative remodeling induced in vitro in both VICs and whole AV leaflets. The beneficial impact of metformin involves modulation of myofibroblastic and osteogenic differentiation as well as ECM remodeling. To the best of our knowledge, this is the first study to (1) show a protective role of metformin in organic phosphate-induced degeneration, and (2) address the limitations of 2D VIC cultures by exploring metformin’s effects in VICs as well as AV leaflets. Overall, our study suggests metformin as a potential candidate for pharmacological treatment to prevent CAVD initiation and progression.

### Study limitations

Our findings are primarily derived from in vitro experiments, thus translation to clinical implications is limited since our 2D experiments focus on VIC monocultures, precluding the complexity of inter-cellular interplay between VIC and other cell types, predominantly valvular endothelial cells. Although we address this issue by including a 3D in vitro CAVD tissue model, this model applies passive tension on AV cusp tissue but without dynamic sheer stress, lacking the influence of hemodynamic forces in a physiological blood system. Furthermore, the induction of degeneration was accomplished by specific culture conditions, which cannot entirely mirror the pathophysiologic in vivo conditions leading to CAVD. This also applies to the supra-pharmacological concentrations of metformin, which exceed the plasma levels achieved in patients. Moreover, the study is limited to ovine cells and tissues, which partially restricts the translational relevance of the findings. This is on the one hand due to restricted access to ‘healthy’ human tissue in sufficient numbers of biological replicates. On the other hand, at this basic stage of investigation, standardized experiments using animal cells and tissues are more feasible, as they are not influenced by patient-related factors such as comorbidities or medication. Nevertheless, results obtained from animal and human in vitro experiments must be interpreted with caution, as they may not fully reflect the complex in vivo processes leading to CAVD. Hence, while our findings provide valuable insights, in vivo studies, especially animal studies ideally involving gender-specific considerations, will have to lead the path towards clinical translation to advance our understanding of metformin and its potential benefit for CAVD treatment.

## Materials and methods

### Preparation of aortic valve leaflets

Aortic valve (AV) leaflets were extracted from the hearts of 6- to 9-month-old sheep (*ovis aries*) derived from a local abattoir (Laame, Wuppertal, Germany). The sheep hearts were obtained as residual by-products from slaughtering procedures, and no animals were sacrificed specifically for research purposes. Accordingly, no experiments involving living animals or humans were performed. AV leaflet preparation was performed as previously described^28^. After removal from the aortic root, AV leaflets were washed repeatedly in cold sterile phosphate-buffered saline (PBS, supplemented with 100 U/mL penicillin-streptomycin (P/S; Thermo Fisher Scientific, MA, US) and 1% Amphotericin B (Gilead Sciences, Foster City, CA, US)) until blood residues were entirely removed.

### Isolation and culture of primary ovine VICs and application of the in vitro CAVD tissue model

Valvular interstitial cells (VICs) were isolated as previously outlined^49^. Concisely, leaflets were extracted, washed in cooled PBS and transferred to Dulbecco’s modified Eagle’s medium containing 4.5 g/L glucose with GlutaMAX supplement (DMEM; Thermo Fisher Scientific, Waltham, MA, US) including 10% fetal calf serum (FCS; Sigma-Aldrich), 100 U/mL P/S, 1% non-essential amino acids (Sigma-Aldrich), and 1 µg/mL amphotericin B (Gilead Sciences). AV leaflets were then cut into small pieces, placed into gelatine-coated cell culture flasks, and cultured at 37 °C with 5% CO_2_ to allow for VIC emigration. Cells in passage 4–6 were used. The in vitro CAVD tissue model was applied by stretching the AV leaflets with needles on silicon rubber rings under passive tension^28^.

### In vitro cultures for degeneration and stimulation

VICs and AV leaflets were cultured under pro-degenerative (pd) conditions (DMEM, 10% FCS, 10 mM β-glycerophosphate disodium salt hydrate (β-GP, Sigma-Aldrich) and 1.5 mM calcium chloride (Sigma-Aldrich). For VIC cultures, cells were seeded upon confluence and then treated for 7 days unless otherwise specified. The medium was changed every 2–3 days. AV leaflets were cultured with 10 mL culture medium up to a cultivation period of 56 days with medium changes once a week. For AV leaflets, experiment readouts were conducted on day 28 unless otherwise specified. In certain experiments, VICs and AV leaflets were incubated with 1,1-Dimethylbiguanide hydrochloride (Metformin; Sigma, Steinheim, Germany; 2–10 mM). After assessment of potential running concentrations, further experiments were conducted with the established concentration of 10 mM metformin.

### Analysis of AV leaflet calcification

Cultivation of AV tissues was followed by photo documentation using a camera (PowerShot SX20 IS, Canon) and a light pad (KAISER slimlite LED). AV leaflets were washed with PBS and photographed on the light pad. Opaque areas were measured with the image analysis software ImageJ version 1.50i (https://imagej.net/).

### Alizarin red S calcium staining of VIC cultures

For alizarin red S calcium staining, VIC monolayers were subsequently washed in PBS, fixed with 4% formalin, and stained with alizarin red S solution for 20 min (pH 4.2; Roth, Karlsruhe, Germany). Observations and photographic records were taken with an inverse microscope system (DM IL Type LED; Leica, Wetzlar, Germany) equipped with a digital camera (DFC425C) using LAS software version 3.8 (https://www.leica-microsystems.com/). For quantification, alizarin red S was extracted for 3 h (shaking, RT) with 100 mM cetylpyridinium chloride monohydrate (Sigma-Aldrich). Absorbance was then measured at 540 nm using a Tecan Infinite M1000 pro microplate Reader (Tecan, Männedorf, Switzerland).

### Calcium content in VIC cultures

For the assessment of calcium content in VIC cultures, a Calcium Assay Kit (Abnova, Taipei City, Taiwan) was utilized according to manufacturer’s instructions. For preparation, cells were rinsed with PBS and lysed in Tris-HCl buffer (0.1 M, pH 7). Absorbance was measured at 612 nm using a Tecan Infinite M1000 pro microplate Reader (Tecan). The total protein content of VIC cultures was determined using the Pierce™ BCA Protein Assay Kit (Sigma-Aldrich) according to manufacturer’s instructions. Calcium content was indexed to total protein content of VIC cultures.

### Alkaline phosphatase and lactate dehydrogenase in supernatants

To quantify the alkaline phosphatase (ALP) content in supernatants an ALP colorimetric assay kit was utilized (Thermo Fisher Scientific) according to manufacturer’s instructions and absorbance was measured at 405 nm. Levels of lactate dehydrogenase (LDH) in supernatants were determined using the LDH Cytotoxicity Assay Kit (Thermo Fisher Scientific) according to manufacturer’s instructions. Absorbance was detected at 490 nm and 680 nm using a Tecan infinite M1000 pro microplate Reader (Tecan).

### Proliferation assay (BrdU)

To assess for cell proliferation of VICs, 5-bromo-2’-deoxyuridine (BrdU) incorporation into cellular DNA was measured using an anti-BrdU antibody with the BrdU Cell Proliferation Assay Kit (Cell Signaling Technologies, MA, US) according to manufacturer instructions. VICs were seeded at a density of 10,000 cells/well in 96-well plates overnight before being treated with metformin for 24 h, 48 h, and 72 h, respectively. Absorbance was detected at 450 nm using a Tecan infinite M1000 pro microplate Reader (Tecan).

### Histological staining: Alizarin red S calcium staining and movat’s pentachrome

After cultivation, AV leaflet tissues were rinsed in PBS, embedded in KP-CryoCompound (VWR Chemicals, Radnor, UK), and then cryopreserved with fluid nitrogen. Cryosections were prepared using a Leica CM1950 microtome and stained with alizarin red S as well as modified Movat’s pentachrome^28^ prior to photographing and analysis. For alizarin red S staining, cryosections were washed with distilled water and stained with a 2% w/v alizarin red S solution (Roth; pH 4.3), followed by dehydration with acetone, acetone-xylene (1:1) and xylene. For quantification, up to 3 slices per individual AV leaflet were imaged and the mean was calculated. Movat’s pentachrome staining was performed as previously described^28^. Sections were sealed with Roti™HistoKitt (Roth) and imaged with a digital camera (Leica DFC 425 C) using a Leica DM2000 microscope.

### ALP staining of VICs and AV leaflet tissue

To determine whether ALP activity is detectable in cultivated cells, treated VICs were incubated with 1-StepTM NBT/BCIP Substrate Solution (Thermo Fisher Scientific) for 30 min at 37 °C in 5% CO_2_ after washing three times with PBS. Images were taken by an inverse microscope system (DM IL Type LED; Leica) equipped with a digital camera (DFC425C) using LAS software version 3.8 (https://www.leica-microsystems.com/). Cells were fixed with 4% formalin. The mean of up to three images per individual experiment was calculated and analyzed. To assess for ALP activity in AV leaflet tissue, colorimetric detection with 1-StepTM NBT/BCIP Substrate Solution (Thermo Fisher Scientific) was performed. Cryosections were incubated with the substrate solution for one hour at 37 °C in 5% CO_2_ and washed with PBS afterwards. Sections were sealed with Leica CV Ultra mounting media (Leica Biosystems) and imaged with a digital camera (Leica DFC 425 C) using a Leica DM2000 microscope. Stained areas were quantified using ImageJ version 1.50i (https://imagej.net/) and calculated as percentage of total area of interest. Up to three sections per AV leaflets were measured and calculated as mean.

### 4’,6-diamidino-2-phenylindole (DAPI) staining

4′,6-diamidino-2-phenylindole (DAPI) staining was performed by fixation of cryosections in 4% formalin for 10 min, incubated in 0.25% Triton-X-100 in PBS for 10 min and washed with PBS three times. Sections were incubated in 4′,6-diamidino-2-phenylindole (DAPI; cat. no.: 6335, Roth) 1:1000 in 1% BSA for 10 min. Then, sections were washed three times with PBS and rinsed in distilled water before sealing with Leica CV Ultra mounting media (Leica Biosystems). Fluorescent micrographs were captured with a DM2000 microscope, a DFC425C camera, and LAS software version 3.8 (Leica). Cell nuclei were quantified using ImageJ version 1.50i (https://imagej.net/) and calculated as amount per total area of interest. Two images per individual experiment were analyzed and the mean was calculated.

### RNA isolation and real time semi-quantitative polymerase chain reaction (RT-qPCR)

Total RNA was isolated from VIC cultures using the RNeasy mini kit (Qiagen, Hilden, Germany) according to manufacturer’s instructions. AV tissue was frozen in liquid nitrogen, crushed with mortar and pestle, and lysed in TRIzol reagent (Thermo Fisher Scientific) prior to purification with the RNeasy mini kit. Reverse transcription was performed using a commercial kit (Quantitect Reverse Transcription Kit, Qiagen) and a Biometra T3000 Thermocycler. Finally, semi-quantitative RT-PCR was conducted using the GoTaq^®^ qPCR Master Mix (Promega, WI, US) on a real-time cycler (Applied Biosystems StepOnePlus; Thermo Fisher Scientific). Primer sequences are shown in Table 1. Ribosomal protein large subunit 29 (RPL29) gene expression was used as a reference gene for normalization using the comparative ΔΔCt method.

**Table 1:** Gene expression analysis primer sequences.

Gene	Forward sequences (5’ – 3’)	Reverse sequences (5’ – 3’)
ACTA2	TAGAACACGGCATCATCACC	TGAGAAGGGTTGGATGCTCT
BMP2	CTTAGACGGTCTGCGGTCTC	GGAAGCAGCAACGCTAGAAG
COL1A1	AAGACATCCCACCAGTCACC	TAAGTTCGTCGCAGATCACG
COL3A1	GACATAGAGGCTTTGATGGACGA	CACTTCCTCGAGCTCCATCG
COL5A1	CGAGAACCCGGATGAGAACC	GGCCTCCGATCCCTTCATAGA
MMP2	TGACAAGGACGGCAAGTATG	GTAAGATGTGCCCTGGAAGC
MMP9	TAGCACGCACGACATCTTTC	GCCCACATAGTCCACCTGAT
BGLAP	GAAGAGACTCAGGCGCTACC	GCTCATCACAGTCAGGGTTG
SPP1	GATGGCCGAGGTGATAGTGT	TCGTCTTCTTAGGTGCGTCA
RPL29	CCAAGTCCAAGAACCACACC	TATCGTTGTGATCGGGGTTT
RUNX2	CTCTGGCCTTCCACTCTCAG	ATGAAATGCTTGGGAACTGC
TGFB1	GAGCCAGAGGCGGACTACTA	TCGGACGTGTTGAAGAACAT
VIM	GACCTGGAGCGTAAAGTGGA	CTCTTGAATCTGGGCCTGAA

*ACTA2* - alpha smooth muscle actin,* BMP2* - bone morphogenic protein 2,* COL1A1 - * alpha- type I collagen,* COL3A1 - * alpha- type III collagen,* COL5A1 - * alpha- type V collagen,* MMP2 - * matrix metalloproteinase 2,* MMP9* - matrix metalloproteinase 9,* BGLAP - * osteocalcin,* SPP1 - * osteopontin,* RPL29 - * ribosomal protein large subunit 29,* RUNX2* -  runt-related transcription factor 2,* TGFB1* - transforming growth factor beta 1,* VIM - * vimentin.

### SDS-PAGE and Western blot analysis

Protein analysis was performed for VIC cultures and AV leaflet tissue samples. Cells were lysed on ice in well-plates using RIPA buffer containing PhosSTOP and cOmplete mini protease inhibitor cocktail (Sigma-Aldrich), homogenized and centrifuged at 14,000 rpm for 20 min at 4 °C. AV leaflet tissue was frozen in liquid nitrogen, crushed with mortar and pestle, and then lysed with RIPA buffer. Total protein content was determined using the DC™ Protein Assay (BioRad). Protein homogenates were separated using the Laemmli method on a 10% reducing SDS- polyacrylamide gel and then transferred to nitrocellulose membranes (BioRad). Protein signals were detected with primary antibodies from Cell Signaling Technology against AMPKα (1:1000, cat. no. 26035) and phospho-AMPKα (1:1000, cat. no. 2535 S). For normalization, primary antibodies against GAPDH (1:2000, cat. no.: C52118) or β-actin (1:2000, cat. no. 4967 L) were used as housekeeper protein signals on the appropriate nitrocellulose membranes. To detect primary antibody signals, HRP-conjugated secondary antibody goat anti rabbit IgG (1:10000, cat. no.: 111-035-003, Dianova) was used. PageRuler Prestained Protein ladder (cat. no.: 26616; Thermo Fisher Scientific) was used for determination of molecular weight. To visualize protein bands, the Western Bright™Quantum Western Blotting Detection System was used (Advansta, Menlo Park, CA, US) according to standard protocols. The membranes were digitalized with an Amersham Imager 600 (GE Healthcare, Freiburg, Germany). Densitometry was analyzed using ImageJ software version 1.50i (https://imagej.net/).

### Statistical analysis and figure preparation

Statistical analysis was performed using Prism 6 software version 6.01 (GraphPad, San Diego, CA; https://www.graphpad.com/). For comparison of two groups, nonparametric Mann-Whitney-U test or Wilcoxon-signed-rank tests were conducted where appropriate. For comparisons including three or more groups, nonparametric Kruskal-Wallis test with Dunn’s multiple comparison post-hoc test was performed. Western blot data with independent variables was analyzed by two-way analysis of variance (ANOVA) with Sidak’s post-hoc test. Data are either reported as standard error of the mean (SEM) and presented as scatter plots with bar graphs or shown as box-and-whisker plot indicating minimum, maximum, and median. Dots in scatter plots represent individual data points. The number of independent replicates for each comparison is indicated in the figure legends. *p-values* < 0.05 were considered significant, and indicated as *p* < 0.05 (*), *p* < 0.01 (**), *p* < 0.001 (***), *p* < 0.0001 (****), respectively. Adobe illustrator (software version 29.1; https://www.adobe.com/) was utilized to create figures.

## Supplementary Information

Below is the link to the electronic supplementary material.

Supplementary Material 1

## Data Availability

The primary data and datasets supporting the findings of this study are available from the corresponding author upon reasonable request.

## Abbreviations

ACTA2: Alpha smooth muscle actin
ALP: Alkaline phosphatase
AMPK: 5’ adenosine monophosphate–activated protein kinase
AV: Aortic valve
BMP2: Bone morphogenic protein 2
BrdU: 5–bromo–2’–deoxyuridine
CAVD: Calcific aortic valve disease
COL1A1: Alpha–type I collagen
COL3A1: Alpha–type III collagen
COL5A1: Alpha–type V collagen
DAPI: Fluorescent 4′,6–diamidino–2–phenylindole
ECM: Extracellular matrix
GAPDH: Glyceraldehyde 3–phosphate dehydrogenase
LDH: Lactate dehydrogenase
MMP: Matrix metalloproteinase
BGLAP: Osteocalcin
SPP1: Osteopontin
pAMPK: Phosphorylated 5’ adenosine monophosphate–activated protein kinase
pd: Pro–degenerative
RUNX2: Runt–related transcription factor 2
TGFB1: Transforming growth factor beta 1
VIC: Valvular interstitial cells
VIM: Vimentin
β-actin: Beta–actin
