# Supplementary material for: Metformin mitigates aortic valve degeneration in an ex vivo three-dimensional tissue model
Source: Sci Rep. 2025 Dec 17;15:44057. doi: 10.1038/s41598-025-31070-6 (PMC12715251; doi:10.1038/s41598-025-31070-6)
Supplement: Supplementary file 1 — Supplementary Material 1 [file 41598_2025_31070_MOESM1_ESM.pdf]

Supplementary Information

Manuscript Title: Metformin mitigates aortic valve degeneration in an ex vivo three-dimensional tissue model

Friederike I. Schoettler[1,2], Andreas Weber[1], Vera Schmidt[1], Sebastian J. Bauer[1,2], Moritz B. Immohr[1,2], Artur Lichtenberg[1], Payam Akhyari[1,2*], Mareike Barth[1,2]

[1]Department of Cardiovascular Surgery, University Hospital Düsseldorf, Heinrich Heine University Düsseldorf, Medical Faculty, Düsseldorf, Germany

[2]Department of Thoracic and Cardiovascular Surgery, Medical Faculty, West German Heart and Vascular Center, University of Duisburg-Essen, Essen, Germany

[*]Corresponding author

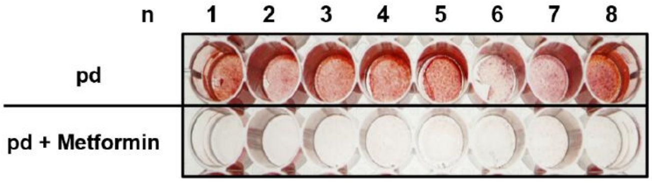

<u>Supplementary Figure S1:</u> Metformin diminishes calcium accumulation in VICs.

Representative macroscopic images of alizarin red S staining showing VIC under pd and pd + metformin treatment (n = 8).

pd – pro-degenerative, VIC – valvular interstitial cell

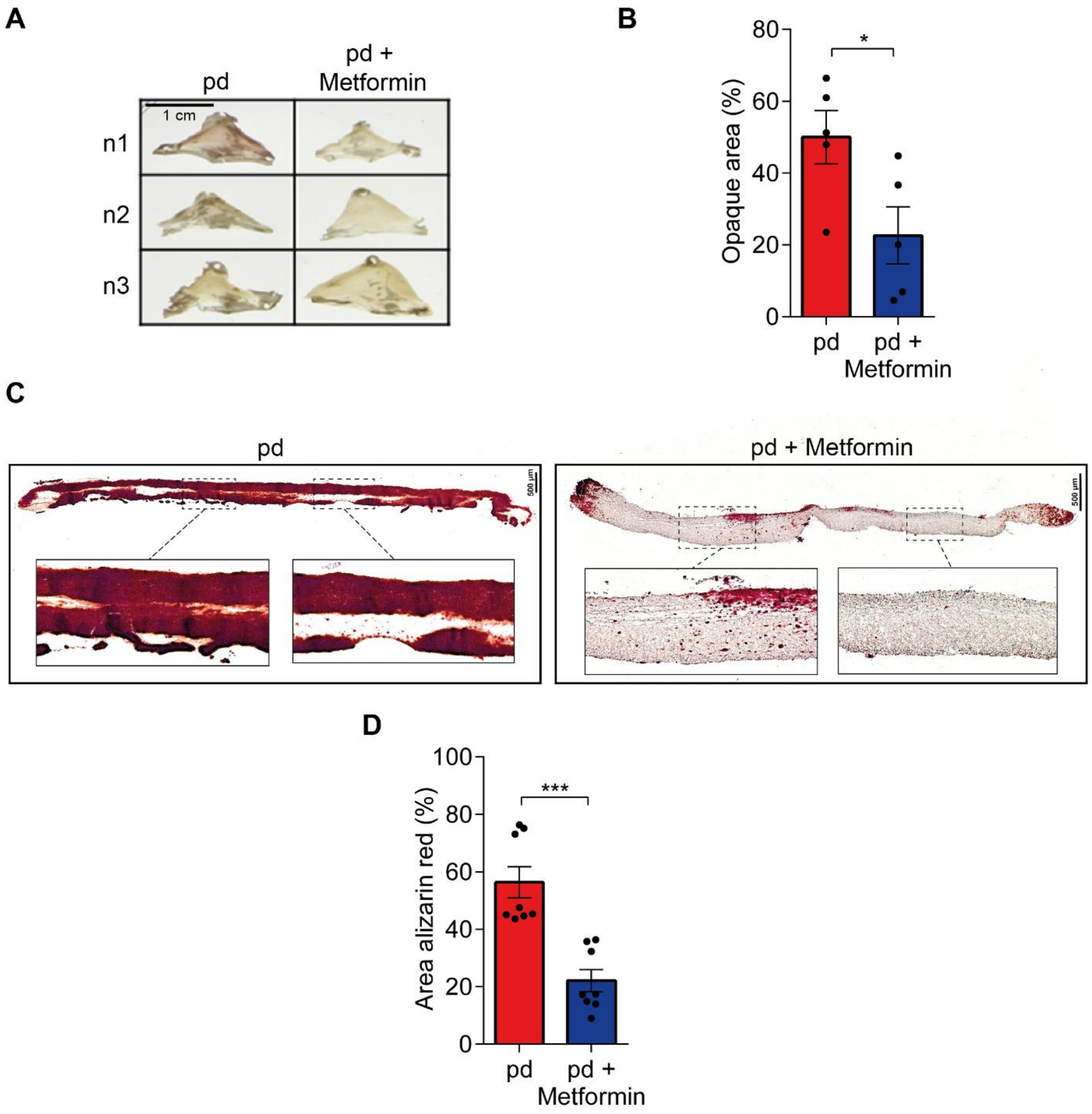

<u>Supplementary Figure S2:</u> Metformin protects AV leaflets against degeneration in long-term culture conditions.

(A) Representative macroscopic images of AV leaflets treated under pd and pd + metformin for 56 days. Three independent experiments are shown imaged on a light pad. Scale = 1 cm. **(B)** Quantification of calcified areas of AV leaflets treated for 56 days presented as percentage of total AV leaflet area (n = 5). **(C)** Representative microscopic images of histological alizarin red S calcium staining of treated AV leaflets with pd (*left*) and pd + metformin (*right*) for 56 days. Scale bar = 500 µm. **(D)** Quantification of alizarin red S calcium-stained areas as percentage of total AV area for pd and pd + metformin (n = 8). p-values were calculated by using nonparametric Mann-Whitney-U test. Data are presented as mean ± SEM. * = $p < 0.05$, *** = $p < 0.001$.

AV – aortic valve, µm – micrometer, pd – pro-degenerative, VIC – valvular interstitial cell

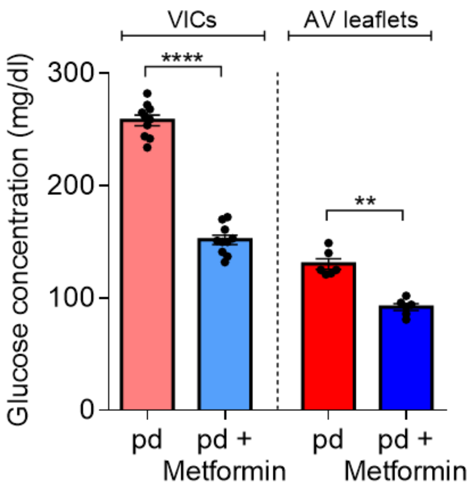

<u>Supplementary Figure S3:</u> Glucose concentrations in culture supernatants of VIC and AV leaflet cultures.

VICs (n = 10) and AV leaflets (n = 6) were treated under pd and pd + metformin conditions for 7 and 28 days, respectively. Glucose concentration in culture media supernatants was measured using a GLUCO Smart Swing blood glucose meter and test strips (MSP Bodmann, Bobingen, Germany). Statistical significance was tested using nonparametric Mann-Whitney-U test. Data are presented as mean ± SEM. ** = $p < 0.01$, **** = $p < 0.0001$.

AV – aortic valve, dl – deciliter, mg – milligram, pd – pro-degenerative, VIC – valvular interstitial cell

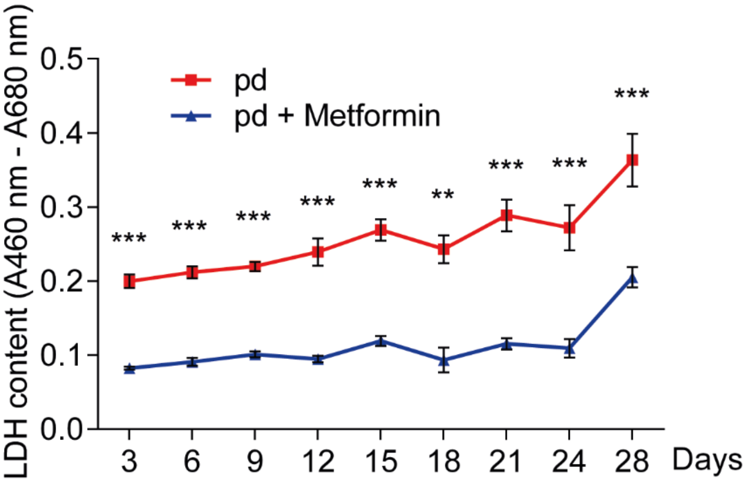

<u>Supplementary Figure S4:</u> **Metformin reduces LDH content in supernatants of the AV leaflet tissue culture model.**

(A) Metformin treatment reduces LDH content in supernatants compared to pd-controls at all timepoints up to 28 days. p-values were calculated by using two-way analysis of variance (ANOVA) with Tukey's post-hoc test. Data are presented as mean ± SEM. ** = $p < 0.01$, *** = $p < 0.001$.

AV – aortic valve, LDH – lactate dehydrogenase, nm – nanometer, pd – pro-degenerative

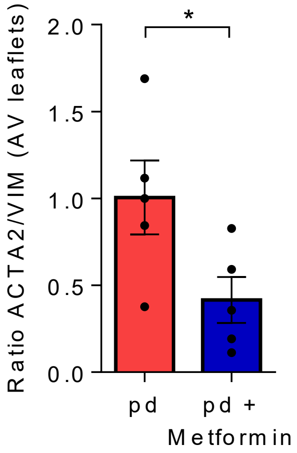

<u>Supplementary Figure S5:</u> Gene expression analysis of AV leaflets assessing the ratio of ACTA2/VIM.

Aortic valve (AV) leaflets were cultured for 28 days under pd and pd + metformin treatment (n = 5). Gene expression analysis was performed to assess for ACTA2 and VIM. The ratio of ACTA2/VIM was calculated. The p-value was calculated by using Mann-Whitney-U test. Data are presented as mean ± SEM. * = $p < 0.05$.

ACTA2 – alpha smooth muscle actin, pd – pro-degenerative, VIM - vimentin

Cropped blots Figure 6A **According uncropped blots Figure 6A**

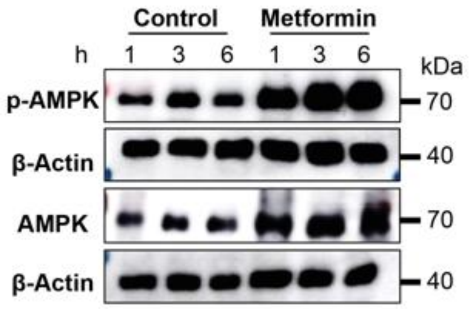

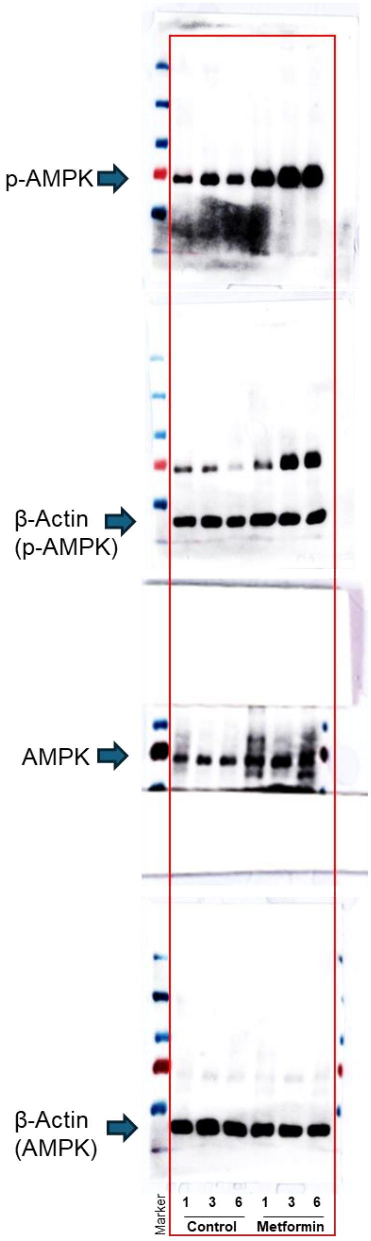

<u>Supplementary Figure S6:</u> **Uncropped Western blot membrane images shown in Figure 6A.**

Representative cropped (*left*) und according uncropped (*right*) Western blot images of VIC cultures for AMPK, phosphorylated AMPK (p-AMPK), and β-actin at 1, 3, and 6 hours under control conditions (10 % culture medium) and metformin treatment. Lanes in red boxes indicate the cropped-out parts for Figure 6A.

AMPK – 5' adenosine monophosphate-activated protein kinase, β-actin – beta-actin, p-AMPK – phosphorylated 5' adenosine monophosphate-activated protein kinase

Cropped blots Figure 6B

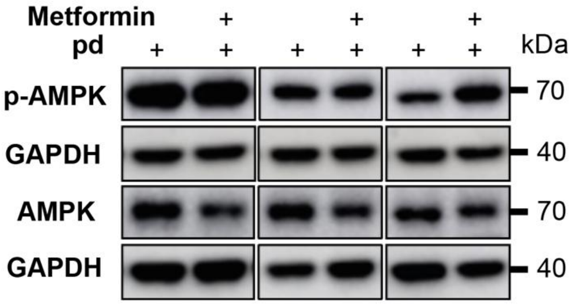

According uncropped blots Figure 6B

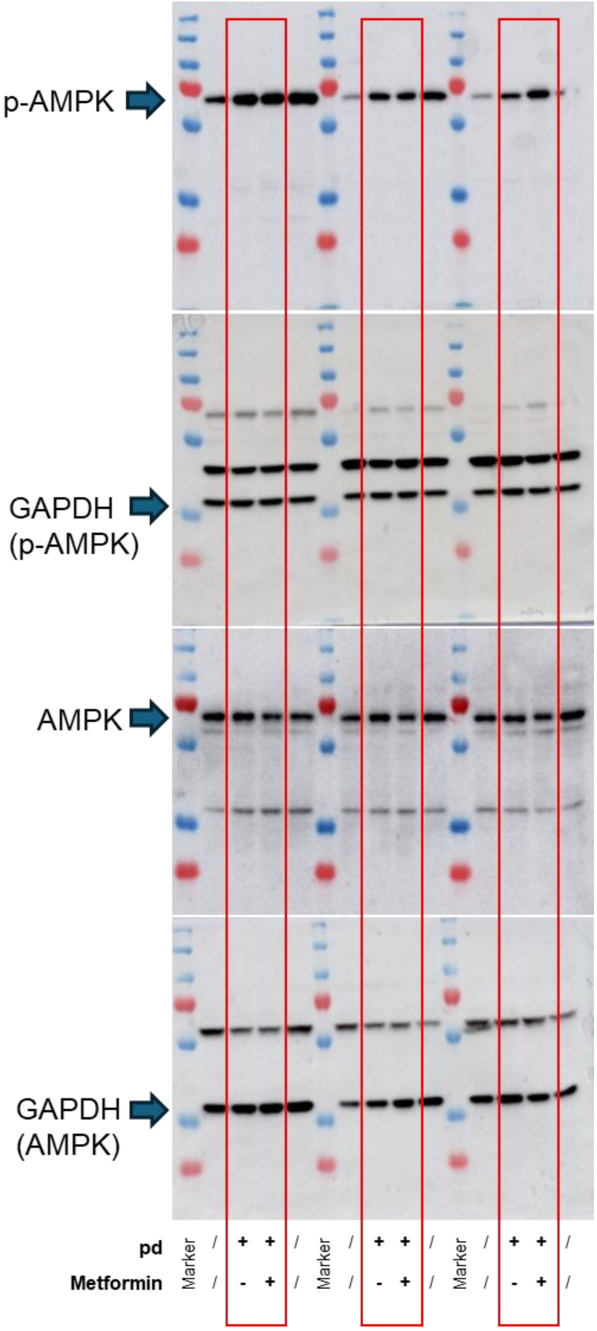

<u>Supplementary Figure S7:</u> Uncropped Western blot membrane images shown in Figure 6B.

Representative cropped (*left*) and according uncropped (*right*) Western blot images of VIC cultures (left) for AMPK, p-AMPK, and GAPDH after 7 days of cultivation under pd and pd + metformin. Lanes in red boxes indicate the cropped-out parts for Figure 6B. The parts of the original images that were not imported in the final figure (shown as: /) consisted of independent experiments that were not included in the contents or analysis of this manuscript.

AMPK – 5′ adenosine monophosphate-activated protein kinase, GAPDH – glyceraldehyde 3-phosphate dehydrogenase, p-AMPK – phosphorylated 5′ adenosine monophosphate-activated protein kinase

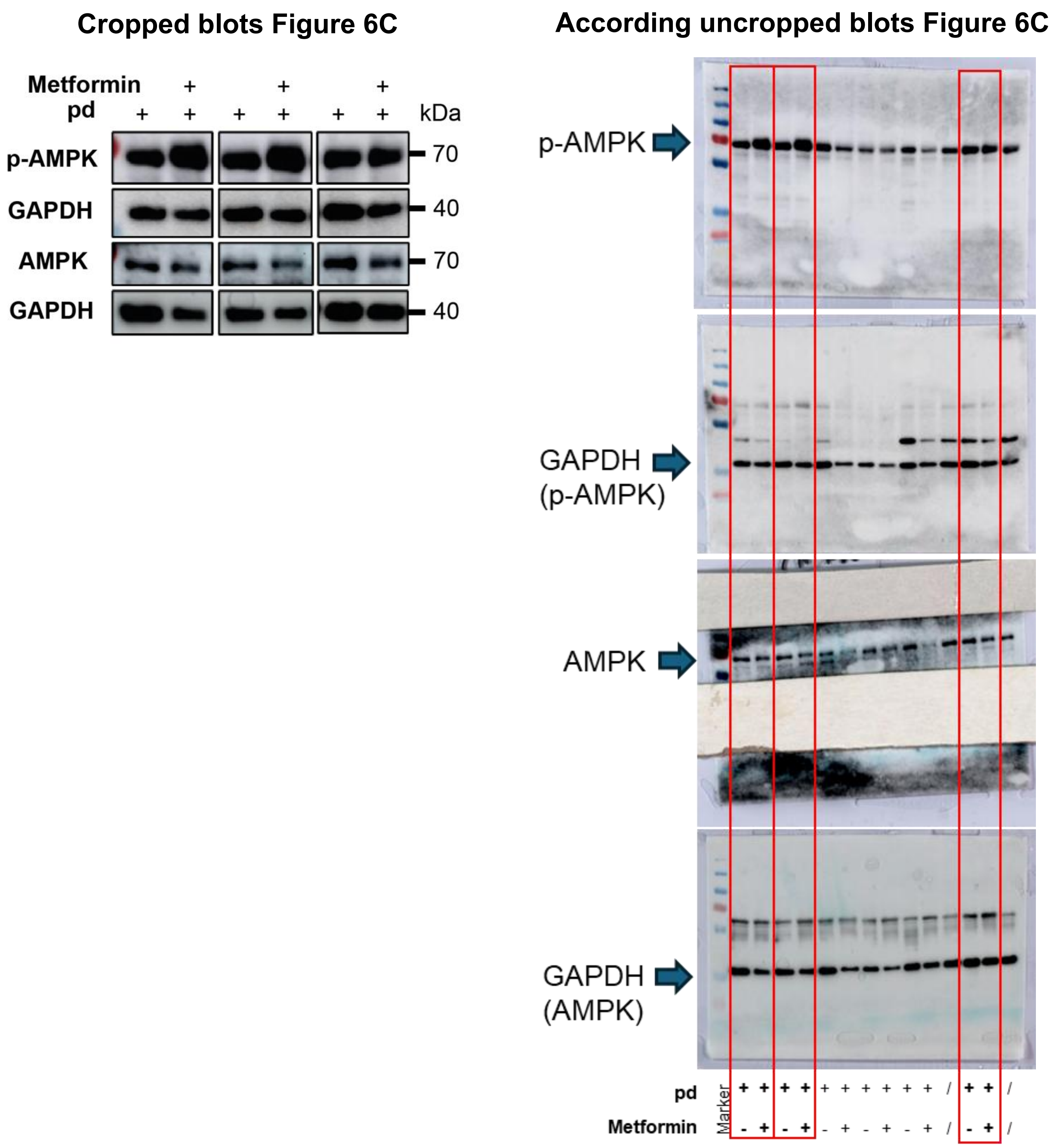

<u>Supplementary Figure S8:</u> Uncropped Western blot membrane images shown in Figure 6C.

Representative cropped (*left*) und according uncropped (*right*) Western blot images of AV leaflets after 28 days of cultivation under pd and pd + metformin for AMPK, p-AMPK, and GAPDH. Lanes in red boxes indicate the cropped-out parts for Figure 6C. Three n were displayed in the final figure for the purpose of representation. The parts of the original images that did not include pd or Metformin conditions (shown as: /) consisted of independent experiments that were not included in the contents or analysis of this manuscript.

AMPK – 5′ adenosine monophosphate-activated protein kinase, GAPDH – glyceraldehyde 3-phosphate dehydrogenase, p-AMPK – phosphorylated 5′ adenosine monophosphate-activated protein kinase
